# Identification of Carotenoids and Isoprenoid Quinones from *Asaia lannensis* and *Asaia bogorensis*

**DOI:** 10.3390/molecules22101608

**Published:** 2017-09-25

**Authors:** Hubert Antolak, Joanna Oracz, Anna Otlewska, Dorota Żyżelewicz, Dorota Kręgiel

**Affiliations:** 1 Institute of Fermentation Technology and Microbiology, Faculty of Biotechnology and Food Science, Lodz University of Technology, 171/173 Wólczańska, 90-924 Lodz, Poland; anna.otlewska@p.lodz.pl (A.O.); dorota.kregiel@p.lodz.pl (D.K.); 2 Institute of Food Technology and Analysis, Faculty of Biotechnology and Food Science, Lodz University of Technology, 4/10 Stefanowskiego, 90-924 Lodz, Poland; joanna.oracz@p.lodz.pl (J.O.); dorota.zyzelewicz@p.lodz.pl (D.Ż.)

**Keywords:** *Asaiabogorensis*, *Asaialannensis*, carotenoids, isoprenoid quinones, phytofluene, neurosporene, canthaxanthin, zeaxanthin, menaquinone, ubiquinone-10

## Abstract

The aim of the study was to identify and quantitatively assess of carotenoids and isoprenoid quinones biosynthesized by six different strains of acetic acid bacteria, belonging to genus *Asaia*, that are common beverage-spoiling bacteria in Europe. Bacterial cultures were conducted in a laboratory liquid culture minimal medium with 2% sucrose. Carotenoids and isoprenoid quinones were investigated using UHPLC-DAD-ESI-MS analysis. In general, tested strains of *Asaia* spp. were able to produce 10 carotenoids and 3 isoprenoid quinones: menaquinone-7, menaquinone-8, and ubiquinone-10. The main identified carotenoids in *Asaia lannensis* strains were phytofluene, neurosporene, α-carotene, while for *Asaia bogorensis*, neurosporene, canthaxanthin, and zeaxanthin were noted. What is more, tested *Asaia* spp. were able to produce myxoxanthophyll, which has so far been identified primarily in cyanobacteria. The results show that *A. lannensis* are characterized by statistically higher concentrations of produced carotenoids, as well as a greater variety of these compounds. We have noted that carotenoids were not only accumulated by bacterial cells, but also some strains of *A. lannensis* produced extracellular carotenoids.

## 1. Introduction

Carotenoids are a group of naturally occurring pigments widely distributed in nature. These compounds are responsible for red, orange, yellow colors of plants, algae, fungi and bacteria. They belong to secondary metabolites, produced in terpenoid biosynthetic pathway [1]. The presence of carotenoids in photoautotrophic organisms as photoprotectants is considered to be a commonly occurring feature. On the other hand, in the heterotrophic microorganisms, biosynthesis of these compounds is relatively rare, and considered as a phenomenon associated with adaptability to the environment [2]. Occurrence of carotenoids have been reported in thermophilic (*Thermus thermophilus*), psychrotrophic (*Arthrobacter agilis*), radioresistant (*Rubrobacter radiotolerans*), halophilic (*Salinibacter ruber*, *Rubrobacter bracarensis*, *Halobacillus litoralis*), and other microorganisms [3,4,5,6,7]. These compounds are characterized by many biological activities. Generally, they are considered to be strong antioxidants, protecting from harmful UV radiation, chemical and salt stress, as well as scavengers of reactive nitrogen species and reactive oxygen species. What is more, carotenoids produced by microorganisms are responsible for membrane stabilization—strengthening membrane integrity [8,9,10]. However, there are still many unknowns about the function of carotenoids in bacterial cells. Functions and regulation of synthesis of carotenoids under different environmental conditions, as well as the impact of these compounds on environmental factors, are areas of interest. Moreover, the greater part of the current research results describes carotenoids from microorganisms inhabiting natural environments, while little is known about these biogenic compounds in industrial microorganisms—especially those contaminating food [1].

Bacteria *Asaia lannensis* and *Asaia bogorensis*, belonging to *Acetobacteraceae* family, are isolated both from natural and industrial environments. So far, they have been found as a microflora inhabiting flowers and fruits in tropical climate [11,12], gut, and the reproductive tract of mosquitos [13], as well as being isolated as opportunistic pathogens from pediatric patients and adults with immunodeficiency [14,15]. Due to the low sensitivity to preservatives and disinfectants, these bacteria are systematically reported as a contamination of functional beverages in Europe, and the primary source of *Asaia* spp. were fruit concentrates used in the production of soft drinks [16,17,18,19]. These bacteria exhibit special abilities of adhesion and biofilm formation on the inner surfaces of industrial installations. In spoiled drinks, they form pink or orange aggregates of cells surrounded by their extracellular polymers. Strains isolated from contaminated products form small, pale, smooth, orange–pink colonies [18]. The presence of *Asaia* spp. in such different environments proves that these bacteria exhibit exceptional cell adaptability. One of the factors facilitating adaptation and growth in diverse environments can be pigments produced by bacterial cells. However, pigments produced by *Asaia* strains have not been identified and characterized, yet. Therefore, the objective of this study was to identify carotenoids synthesized by six different strains of *Asaia* spp. isolated from commercial spoiled soft drinks in Poland.

## 2. Results and Discussion

### 2.1. Carotenoids Identification

**Figure A1:**
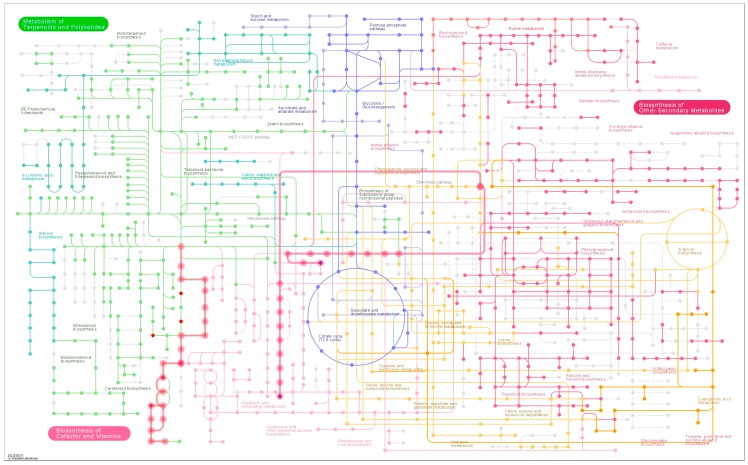
Biosynthesis of secondary metabolites (carotenoids and isoprenoid quinones) by *Asaia lannensis* and *Asaia bogorensis.* Bolded pink lines with red markers are carotenoids. Bolded pink lines with purple markers are ubiquinone-10 and menaquinone.

The characterization and quantitation of the compounds isolated from six strains of *Asaia bogorensis* and *Asaia lannensis* were carried out by UHPLC-DAD-ESI-MS analysis. As a result, 13 major compounds were identified and marked as peaks 1–13 (Table 1). The majority of isolated compounds were classified into a group of carotenoids and designated as peaks from **1** to **9**, and peak **11**, while other compounds (peaks **10**, **12** and **13**) were isoprenoid quinones (Appendix A Figure A1).

**Figure 1:**
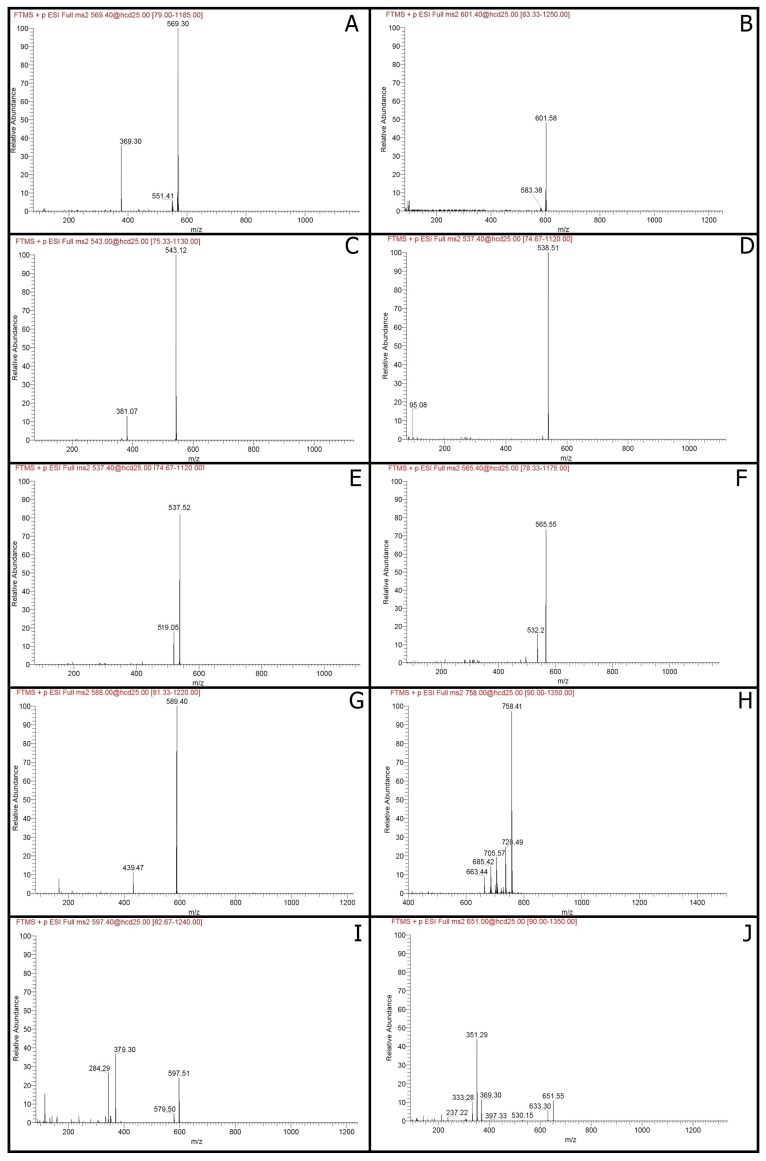
Mass spectra obtained using UHPLC-DAD-ESI-MS. (**A**) zeaxanthin; (**B**) neoxanthin; (**C**) phytofluene; (**D**) neurosporene; (**E**) α-carotene; (**F**) canthaxanthin; (**G**) synechoxanthin; (**H**) myxoxanthophyll; (**I**) astaxanthin; and (**J**) menaquinone-7.

Zeaxanthin (peak **1**) was identified based on a positively charged molecular ion ([M − H]^+^) at *m*/*z* 569.4788, and with fragment ions MS2 at 551 and 369 (Figure 1A). Peak **2** exhibited a [M − H]^+^ at *m*/*z* 601.5769 and MS2 fragment at *m*/*z* 583 was identified as neoxanthin (Figure 1B). Peak **3** had a [M − H]^+^ at *m*/*z* 543.1193 which yielded MS2 fragment at *m*/*z* 381 and was identified as phytofluene (Figure 1C), while peak **4** owned a molecular ion peak [M − H]^+^ at *m*/*z* 538.5083 with MS2, yielding fragment at *m*/*z* 95, which might indicate the presence of neurosporene (Figure 1D). α-Carotene (Figure 1E) with [M − H]^+^ at *m*/*z* 537.5193 and fragment ion MS2 equal *m*/*z* 519, was designated as peak **5**, while β-carotene with similar mass (537.3791) and different MS2 fragment ions (*m*/*z* 444, 333, 177) was designated as peak **11**. Further compounds were canthaxanthin (*m*/*z* 565.5489; MS2 fragment *m*/*z* 532) designated as peak **6** (Figure 1F), synechoxanthin (*m*/*z* 589.3875; and MS2 fragments *m*/*z* 439, 163) designated as peak **7** (Figure 1G). Peak **8** yielded a [M − H]^+^ at *m*/*z* 758.4098 and MS2 fragments at *m*/*z* 728, 705, 685, 633 was identified as myxoxanthophyll (Figure 1H), while peak **9** with molecular ion peak [M − H]^+^ at *m*/*z* 597.5089 and fragment ions equaled *m*/*z* 579, 379 and 285 was identified as astaxanthin (Figure 1I).

**Figure 2:**
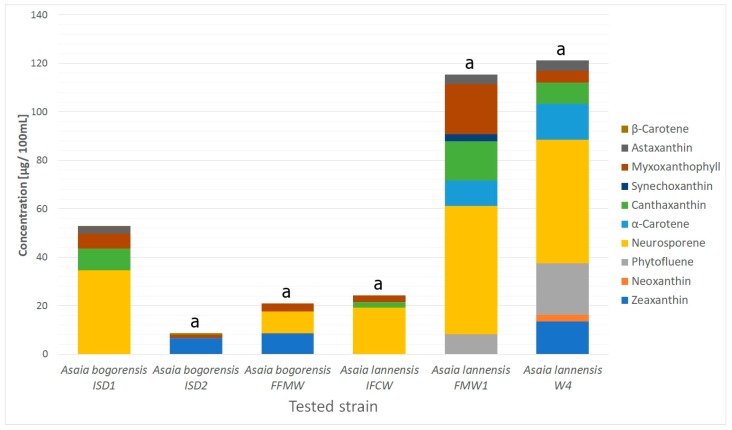
Profiles of carotenoids produced by *Asaia* spp. Values are statistically different (*p* < 0.05). a: *p* < 0.005. The results were compared to those received for *Asaia bogorensis* ISD1.

The highest concentration of carotenoids was found for *A. lannensis* strains W4, and the mean value of these compounds equaled to 121.3 ± 7.56 µg per 100 mL (Figure 2). A slightly lower value of 115.3 ± 6.56 µg/100 mL was obtained for *A. lannensis* FMW1. The lowest concentrations were determined for *A. bogorensis* ISD2 (7.8 ± 0.16 µg/100 mL), and *A. bogorensis* FFMW (20.8 ± 0.81 µg/100 mL). The obtained profiles of biosynthesized carotenoids showed statistically significant differences both at the species and strain levels. Comparative results indicate that carotenoids are produced in higher concentration by *Asaia lannensis* strains. Besides quantitative differences, the results showed qualitative differences. *Asaia lannensis* strains were characterized by a greater variety of synthesized carotenoids. Again, *A. lannensis* W4, exhibited greater diversity, producing 8 out of 10, while *A. lannensis* FMW1 synthesized 7 out of 10 carotenoids identified for *Asaia* spp. On the other hand, *A. bogorensis* ISD1 was characterized by the highest diversity (5 out of 10 carotenoids) from the tested strains of the *Asaia bogorensis* species.

**Figure A2:**
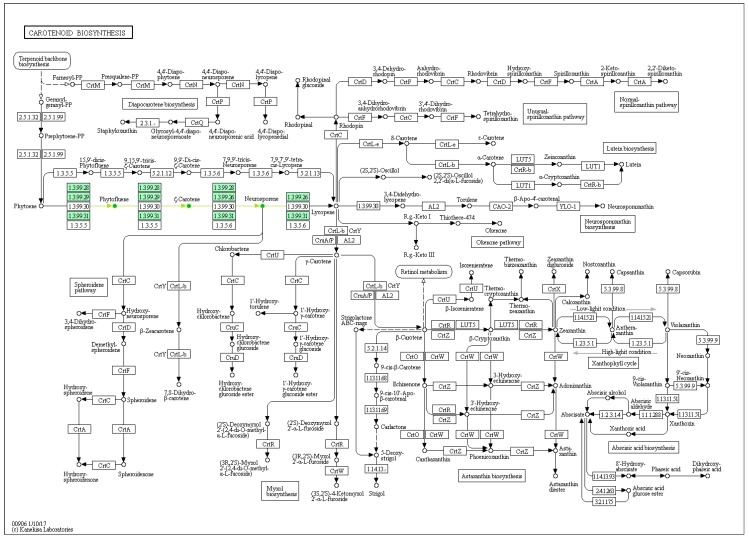
Genes encoding carotenoids identified for *Asaia bogorensis* (marked in green) by Kawaii et al. (2015) [20]. Adapted from Kawaii et al. (2015) using KEEG mapper. The pathway of phytofluene and neurosporene synthesis is green lines with dark-green markers.

**Figure A3:**
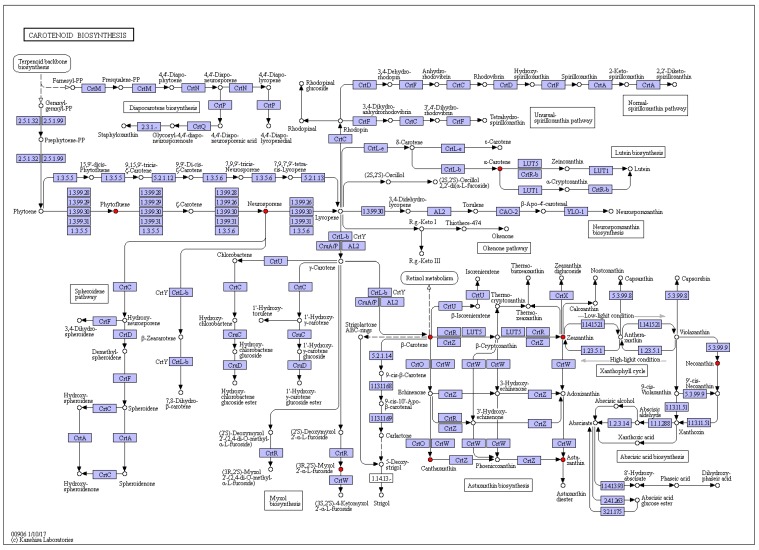
Carotenoid produced by *Asaia lannensis* and *Asaia bogorensis* strains tested in conducted study. Carotenoids are marked in red.

In studies conducted by Kawaii et al. (2015) it was found that bacterium *Asaia bogorensis* has an operon with genes that were homologous to rhodopsin and beta-carotene 15,15′-monooxygenase—participating in the synthesis of phytofluene and neurosporene (Appendix A Figure A2) [20]. Our results showed that tested strains of *A. bogorensis* did not accumulate the first compound, but can produce neurosporene. Generally, the results suggest that *A. bogorensis* have abilities of β-carotene, zeaxanthin, canthaxanthin, and astaxanthin production (Appendix A Figure A3). However, differences between strains were noticeable at the levels of produced carotenoids and the pathways which they take part in. In the case of *Asaia bogorensis* ISD1, we have noted the presence of β-carotene and canthaxanthin, which are the intermediates of astaxanthin production. On the other hand, β-carotene can be used to zeaxanthin biosynthesis, and this was noted for *A. bogorensis* ISD2. It is interesting that astaxanthin can be biosynthesized both from β-carotene via zeaxanthin and adonixanthin. In parallel, zeaxanthin can be used in the pathway of neurosporene production (noted for both species). It is noteworthy that the distinctive feature of *A. bogorensis* from *A. lannensis* is the production of α-carotene and lack of β-carotene production by *A. lannensis*. Thus, the probable biosynthesis of astaxanthin is similar to that of *A. bogorensis*, excluding the use of β-carotene for zeaxanthin or canthaxanthin production. However, in order to confirm the assumptions concerning the activities of proposed pathways, it is necessary to conduct detailed research on the metagenomics of these bacteria.

The initial stage of the biosynthesis of carotenoids belongs to non-mevalonate pathway (MEP), also known as the isoprenoid pathway. In general, carotenoid biosynthesis is catalyzed by a number of enzymes, such as geranylgeranyl pyrophosphate (GGPP) synthase, phytoene synthase, carotene desaturase, and lycopene cyclase. Modification of carotenes is further catalyzed by β-carotene ketolase and β-carotene hydrolase, to generate various C-40 carotenoids [1]. The precursor of carotenoids, geranylgeranyl pyrophosphate (GGPP), is synthesized from farnesyl pyrophosphate (FPP) and isopentenyl diphosphate (IPP). This is followed by condensation of two molecules of GGPP by phytoene synthase (encoded by *CrtB* genes), and generation of phytoene-precursor of C-40 carotenoids. Next, phytoene is desaturated to neurosporene or lycopene via phytofluene by phytoene desaturase (encoded by *crtI* genes) [21]. The results of UHPLC-DAD-ESI-MS analysis performed in our study showed that *Asaia* spp. were able to produce and accumulate neurosporene, thus, phytoene desaturase performs three successive desaturations. In turn, colorless phytofluene was identified in only two strains of *Asaia lannensis*. It is assumed that the content of neurosporene in carotenogenic organisms is very low, while our study suggest that this compound is the main carotenoid produced by *Asaia* spp. (from 42 to 79% of total carotenoids). This strong antioxidant and UV radiation protectant also occurs in other Gram-negative bacteria belonging to genera from α-*Proteobacteria*: *Allochromatium*, *Rhodobacter*, as well as *Rhodovulum* [22,23]. In *Rhodobacter* and *Rhodovulum*, the process of desaturation of phytoene is stopped at neurosporene, instead of proceeding to the usual product, lycopene. Considering this, it can be stated that a similar process occurs in the bacteria *Asaia lannensis* and *Asaia bogorensis*.

The further process of carotenoid biosynthesis involves lycopene cyclization, which is an isomerization reaction that can occur at one or both ends of the carotenoid molecule. This transformation can lead to production of monocycylic γ-carotene, which can be transformed in monocyclic chlorobactene or dicyclic β-carotene, as well as dicyclic myxoxanthophyll derivatives. Generally, lycopene cyclases (CrtY, CrtL, cruA) are members of the FixC protein superfamily. β-Cyclases of the CrtY subfamily are found in Gram-negative and Gram-positive bacteria, such as *Paracoccus haeundaensis* and some *Chloroflexi* phylum [21]. However, based on the obtained results, we are not able to conclude that they are involved in β-carotene synthesis in *Asaia bogorensis* ISD1 and ISD2. It seems that in the case of *Asaia* strains, direct cyclization to myxoxanthophyll or β-carotene occurs. In general, due to the presence of β-ionone ring in the molecule of myxoxanthophyll, monocyclic γ-carotene is presumed to be an intermediate. It is believed that, in the synthesis of this compound, lycopene cyclases are involved. Depending on the strain of microorganism, different lycopene cyclases are involved in different microorganisms. For example, in *Synechococcus* sp. and *Synechocystis* sp., these are cruA and cruP, while for *Synechococcus* sp. strains PCC 7942 and PCC 6301, CrtL and cruP homologs were reported. Derivatives of myxol have been reported for Gram-negative, orange-colored bacteria *Robiginitalea myxolifaciens*, *Gemmatimonas aurantiaca*, as well as bacteria belonging to *Flavobacterium* genus [24,25,26]. Furthermore, it is believed that myxoxanthophyll is a unique glycosylated carotenoid occurring almost exclusively in cyanobacteria. The results of our research show that this compound can be synthesized by both species of *Asaia*, *A. bogorensis* and *A. lannensis*. Thus, we have shown that the unusual acetic bacteria belonging to the genus *Asaia* are also characterized by the biosynthesis of this compound.

In subsequent stages, the β-carotene is transformed by addition of two keto groups to canthaxanthin. The enzyme involved in this reaction in Gram-negative bacteria, such as *Agrobacterium aurantiacum*, *Bradyrhizobium* sp., *Brevundimonas* sp., and *Paracoccus* sp., is β-C-4-oxygenase (β-carotene-4,4-ketolase) type CrtO or CrtW. In our study, canthaxanthin was produced by *Asaia bogorensis* ISD1, which simultaneously produced β-carotene. On the other hand, β-carotene can also be an intermediary in the production of zeaxanthin, which is biosynthesized by 3- and 3′-hydroxylation of β-carotene conducted by beta-carotene 3-hydroxylase (encoded by *crtZ* genes) and β-carotene hydroxylase (encoded by *crtR* genes) [27]. Bacteria synthesizing this carotenoid are strains of *Erwinia herbicola* (syn. *Pantoea agglomerans*)*, Dunaliella salina*, as well as *Flavobacterium* sp., *Neospongiococcum* sp., and *Synechocystis* sp. [28]. What is interesting, considering the fact that *Asaia* spp. produces both canthaxanthin and zeaxanthin, it can be concluded that they use these compounds as intermediates in the biosynthesis of red-colored astaxanthin. The biosynthesis of astaxanthin from zeaxanthin requires only the addition of carbonyl moieties at the C4 positions of the β-rings of zeaxanthin, with the participation of β-carotene ketolase (CrtW type). Whereas the second one would begin with the oxidation of β-carotene, and would have echinenone, canthaxanthin, and adonirubin (phoenicoxanthin) as intermediates. CrtZ proteins, responsible for these reactions, derived from α-*Proteobacteria*, γ-*Proteobacteria*, and cyanobacteria, have been reported so far [29]. This statement can be confirmed by the results obtained for *Asaia bogorensis* ISD1, which produced β-carotene, canthaxanthin, and finally, astaxanthin. On the other hand, *A. lannensis* W4 demonstrated the ability of zeaxanthin and astaxanthin production. The results obtained for all strains suggest that the biosynthesis process is a strain-dependent feature. In order to determine the specific pathways of biosynthesis of these compounds in *Asaia lannensis* and *Asaia bogorensis*, it is necessary to carry out detailed studies on these bacteria.

**Figure 3:**
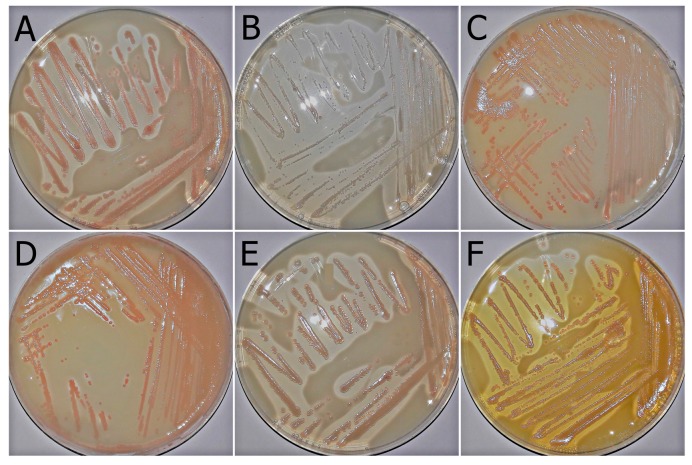
Growth of tested strains on GC agar medium. (**A**) *Asaia bogorensis* ISD1; (**B**) *A. bogorensis* ISD2; (**C**) *A. bogorensis* FFMW; (**D**) *Asaia lannensis* IFCW; (**E**) *A. lannensis* FMW1; (**F**) *A. lannensis* W4. Under the influence of the produced organic acids, the color of the medium is changed from creamy to transparent. In addition, the *Asaia lannensis* strain W4 was characterized by the ability to synthesize extracellular carotenoids, which is evidenced by the change in color of the medium to orange.

The results of quantitative measurements of carotenoids are in the line with the macroscopic observations of the growth of these bacteria on the GC agar medium (Figure 3). Clearly visible were the pink, pale-pink colonies in the case of almost all tested strains. The exception was *Asaia bogorensis* ISD2, whose colonies were creamy (Figure 3B). Moreover, an extremely interesting case is the *A. lannensis* W4, which achieved the highest value of intracellular carotenoids, and in addition, showed the abilities of the production of extracellular carotenoids (Figure 3F). This is evidenced by the color change of the GC agar medium, that before inoculation was creamy-white. It is worth noting that our study is the first report of the extracellular carotenoids biosynthesis by bacteria belonging to genus *Asaia*. Moreover, in the case of other microorganisms, biosynthesis of extracellular carotenoids is not a commonly occurring feature, and has been described in *Deinococcus radiodurans*—an extremophilic, radiation-resistant bacterium [30]. It is possible that in the case of *Asaia lannensis* W4, this feature is combined with the capabilities of other extracellular compound production, such as polysaccharides, which have been noted in the work of Kręgiel (2013) [31]. What is more, as it was mentioned above, this strain was characterized by the highest production of intracellular carotenoids. It is also possible that due to the greater permeability of cell membranes, occurring due to environmental conditions (temperature, calcium ions, UV, and carotenoids themselves), the excess carotenoids are excreted into the environment [32]. Despite the lack of specific mechanisms responsible for the biosynthesis of extracellular carotenoids by *Asaia lannensis* W4, it should be noted that the strain is an unusual case of bacterium, arousing interest. From the point of view of functional beverage contamination, extracellular carotenoids can cause increased organoleptic changes. On the other hand, strains showing, extra- and intracellular carotenoid production are of interest, due to the medical, pharmacological, and food uses of these compounds.

### 2.2. Isoprenoid Quinones Identification

**Figure A4:**
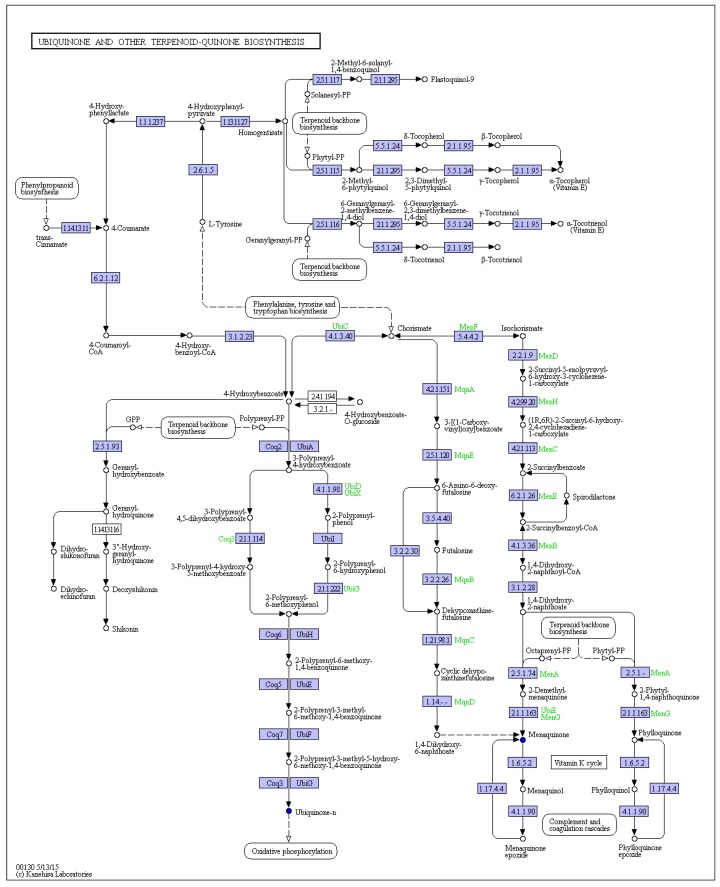
Isoprenoid quinones (ubiquinone-10 and menaquinones) produced by *Asaia lannensis* and *Asaia bogorensis*. Isoprenoid quinones are marked in blue.

Our results show that *Asaia lannensis* and *Asaia bogorensis* are characterized by the ability of ubiquinone-10 and menaquinones biosynthesis (Appendix A Figure A4). The results of chromatographic analysis showed that peak **10** (Figure 1J) had the molecular ion [M − H]^+^ at *m*/*z* 651.5496, and MS2 fragments at *m*/*z* 633, 397, 369, 351 was menaquinone-7 (MC-7), while peak **11** (*m*/*z* 717.4471; MS2 fragments *m*/*z* 575, 187) was characterized as menaquinone-8 (MC-8). Both of these compounds belong to isoprenoid quinones, and are classified as derivatives of a lipo-soluble vitamin K. Menaquinones can be found in many groups of bacteria, such as γ-*Proteobacteria*, δ-*Proteobacteria*, ε-*Proteobacteria*, green sulfur bacteria, green filamentous bacteria. These compounds were noted in the work of Kaiser et al. (2012). In their work, MC-7 was identified in *Arthrobacter nicotianae*, *Brevibacterium linens*, *Micrococcus luteus*, *Rhodococcus equi*, while MC-8 was found in *Arthrobacter bergerei, Arthrobacter protophormoniae*, *Arthrobacter uratoxydans*, *A. nicotianae*, *B. linens*, *M. luteus*, and *R. equi* [33]. It is worth noting that these microorganisms are isolated from extreme environments, such as oil, brine, mural paintings, clinical specimen, sewage, and activated sludge, and also can cause chronic infections [34,35]. In bacterial cells, they are constituents of plasma membranes, playing an essential role in active transport, electron transport, and oxidative phosphorylation [36]. The reduced menaquinones can be rapidly oxidized in the presence of oxygen, therefore, these compounds cannot efficiently operate in an atmosphere containing oxygen [37]. It is believed that menaquinones have undergone transition to other quinones with a higher reduction potential, due to the Earth’s atmosphere changes and the appearance of oxygenic photosynthesis. Besides the role in microbial respiration, menaquinones exhibit antioxidant properties, and can play a role in protecting cellular membranes from lipid oxidation [38]. What is more, it has been noted that menaquinones are involved in the biofilm formation. In the work of Berenjian et al. (2013), authors noted a linear correlation between menaquinone-7 synthesis and *Bacillus subtilis* biofilm formation [39]. Similar results have been obtained in studies on other strains belonging to *B. subtilis* [40]. In our study, all tested bacteria belonging to *Asaia lannensis* and *Asaia bogorensis* produced menaquinone-7, and the highest content was noted to *A. lannensis* FMW1. Moreover, *Asaia lannensis* strains were characterized by statistically higher production of this compound compared to *A*. *bogorensis*. Taking into account that all of the tested strains of *Asaia* bacteria are characterized by the ability to produce menaquinone-7, and the fact that their strong adhesive ability has been demonstrated [18,19] it can assume that menaquinone-7 production can be one of the factors contributing to adhesion and biofouling.

Like menaquinones, ubiquinone-10 (Q-10) (peak **13**) at *m*/*z* 863.6652 and MS2 fragment ion 197, is classified into respiratory isoprenoid quinones, occurring in the plasma membrane of prokaryotes. In contrast to menaquinones that are characterized by low midpoint potentials and are involved in anaerobic respiration, ubiquinones have a higher midpoint potential and are involved in aerobic respiration. Generally, the biosynthesis of ubiquinone is a highly conserved pathway, which begins with production of 4-hydroxybenzoate from chorismite, and in the next steps, involves a large number of genes, named *ubi* (*ubiC*, *ubiA*, *ubiD*, *ubiI*, *ubiG*, *ubiH*, *ubiE*, *ubiF* and *ubiG*). The results of the used method are in line with these obtained by Yamada et al. (2000) and Malimas et al. (2008) [11,41]. In their research, *Asaia bogorensis* and *Asaia lannensis* strains were characterized by occurrence of Q-10 as a quinone system. Kaiser et al. (2012) noted ubiquinone-10 for *Exophiala dermatitidis*, *Filobasidium floriforme*, *Phaffia rhodozyma*, and *Rhodotorula* spp. [33]. What is more, it is said that the majority of the Gram-negative, aerobic rods contain ubiquinones exclusively. For instance, representatives of *Pseudomonas* and *Alcaligenes* genera contain ubiquinones with nine isoprene units (Q-9), while Q-10 predominates in *Agrobacterium* and *Brucella* [42]. In turn, acetic acid bacteria, to which *Asaia* spp. belongs, are characterized by differences in the number of isoprene units contained in their ubiquinone. Like *Asaia*, *Gluconacetobacter* and *Gluconobacter* contain mainly the Q-10-type ubiquinone, while bacteria belonging to genus *Acetobacter* is characterized by the presence of Q-9-type ubiquinone [43]. Furthermore, the content of mixture of menaquinones and ubiquinones can be influenced by the degree of aeration. It was noted that in the case of *Escherichia coli*, low oxygen concentrations increase the level of menaquinones, and therefore, reduce the amount of ubiquinones [42]. On the other hand, Kawai et al. (2015), in the study on *Asaia bogorensis*, found that *A. bogorensis*, like *Escherichia coli*, contains *cyd* genes (encoding cytochrome d oxidase), whose expression is upgraded under oxygen limited conditions [20]. The presence of MC-7 and MC-8, as well as expression of *cyd* genes, is important, due to the fact that *Asaia lannensis* and *Asaia bogorensis* contaminate functional beverages containing low levels of oxygen. It can therefore be presumed that menaquinones and cytochrome d oxidases can synergistically act under conditions of low oxygen concentration, promoting cells survival.

## 3. Materials and Methods

### 3.1. Materials

#### 3.1.1. Bacterial Strains

Six strains of bacteria *Asaia* spp. isolated from fruit-flavored mineral waters and isotonic drinks were used in the study—*Asaia bogorensis* ISD1 (GenBank KP234014), *A. bogorensis* ISD2 (GenBank KP234015), *A. bogorensis* FFMW (GenBank KC756841), *A. lannensis* IFCW (GenBank KP234011), *A. lannensis* FMW1 (GenBank HQ917850), *A. lannensis* W4 (GenBank MF777040). These bacteria were identified using morphological, physiological, and molecular methods, and the nucleotide sequences of 16S rRNA genes were deposited in GenBank (NCBI) [18]. Bacterial strains were deposited in the Pure Culture Collection of Industrial Microorganisms LOCK 105, at the Institute of Fermentation Technology and Microbiology, Lodz University of Technology (Łódź, Poland).

#### 3.1.2. Chemicals and Standards

Acetone and methanol used for the extraction of carotenoids and isoprenoid quinones were purchased from Stanlab (Lublin, Poland). HPLC grade methanol and methyl *tert*-butyl ether (MTBE) were purchased from J.T. Baker (Deventer, The Netherlands). Ultra-pure water was obtained from a Milli-Q water purification system (Millipore Corp., Bedford, MA, USA). Chromacol PTFE syringe filters (0.2 μm pore size) were purchased from Shim-Pol (Izabelin, Poland).

### 3.2. Methods

#### 3.2.1. Culture Conditions

*Asaia lannensis* and *Asaia bogorensis* strains were pre-cultured in 10 mL of liquid GC medium (2% (*w*/*v*) glucose, 0.3% (*w*/*v*) peptone, 0.3% (*w*/*v*) yeast extract, 0.7% (*w*/*v*) CaCO_3_) at 25 °C for 48 h. Then, flasks with 99 mL of sterile liquid minimal medium (2% sucrose (*w*/*v*), 0.3% (NH_4_)_2_PO_4_ (*w*/*v*), 0.3% KH_2_PO_4_ (*w*/*v*), 0.3% MgSO_4_ × 7H_2_O (*w*/*v*), 0.05% (*w*/*v*) yeast extract) were inoculated with 1 mL of standardized bacterial suspensions, in order to obtain a final concentration 10^5^–10^6^ cell per mL. According to our previous studies, these media are suitable for the growth of *Asaia* spp. [44]. Bacterial cultures were incubated for 14 days at 25 °C in the laboratory incubator with the access to sunlight. Bacterial cultures were transferred to 50 mL Falcon tubes, and cells were harvested by centrifugation at 6500 rpm for 10 min at 4 °C, washed with PBS solution (pH 7.4), and stored at −20 °C, until use.

#### 3.2.2. Extraction of Carotenoids

Frozen cell pellets were thawed, and 10 times volume of acetone/methanol (7:3, *v*/*v*) was added. Extractions were carried out at 60 °C for 2 h at laboratory shaker (200 rpm) in darkness. Subsequently, residues of bacterial biomass were harvested by centrifugation at 6500 rpm for 10 min at 4 °C. The obtained supernatant was frozen −20 °C until chromatographic analysis of carotenoids and isoprenoid quinones.

#### 3.2.3. UHPLC-DAD-ESI-MS Analysis

Thawed extracts were analyzed to determine their carotenoids profiles using UHPLC + Dionex UltiMate 3000 system (Thermo Fisher Scientific Inc., Waltham, MA, USA), coupled to both a diode array detector with multiple-wavelengths (Thermo Fisher Scientific Inc., Waltham, MA, USA) and a Q-Exactive Orbitrap™ mass spectrometer (Thermo Scientific, Hudson, NH, USA). Instrument control, data acquisition, and evaluation, were done with the Qexactive Tune 2.1, Chromeleon 6.8 Chromatography Data System, and Thermo Xcalibur 2.2 software (Thermo Fisher Scientific Inc., Waltham, MA, USA), respectively. Chromatographic separation was achieved on a Accucore C30 column (100 mm × 3.0 mm i.d., 2.6 μm; Thermo Fisher Scientific Inc., Waltham, MA, USA) maintained at 30 °C. A ternary solvent system, comprising water as solvent A, methanol as solvent B, and MTBE as solvent C, was used under gradient mode according to the method of Zheng et al. (2017), with some modifications [45]. The initial mobile phase (0.35 mL·min^−1^) was 90% B and 5% C, increased to 95% B (in 12 min), changed to 89% B and 11% C (over 13 min), to 75% B and 25% C (over 15 min), to 50% B and 50% C (in 20 min), followed by a reconditioning at initial conditions for 2 min. The sample injection volume was 5 µL. Chromatograms were recorded at 280, 450, 486 and 520 nm. Mass spectral data were collected in the positive ionization mode with an electrospray source. The mass spectrometer conditions were as follows: capillary temperature was 250 °C; heater gas temperature was set at 400 °C; electrospray capillary voltage was 3.5 kV. The nebulizer gas and collision gas was nitrogen. The collision energy was 25 eV. Full-scan MS and target MS2 spectra were obtained by scanning *m*/*z* from 200 to 1000. Instrument control, data acquisition and evaluation were done with the Qexactive Tune 2.1, Chromeleon 6.8 Chromatography Data System, and Thermo Xcalibur 2.2 software, respectively. Identification and peak assignment of carotenoids were based on the comparison of their retention times, UV–visible absorbance spectra characteristics, full scan mass spectra, and MS/MS fragmentation patterns with those of authentic standards analyzed under identical conditions, as well as the bibliographic references used in the characterization process [33,46]. Quantification of individual compounds was carried out using external standard method.

### 3.3. Statistics

Three independent experiments were performed, and from the obtained data, means with standard deviations were calculated. Statistical differences between the obtained carotenoid profiles were compared using a one-way analysis of variance (ANOVA; OriginPro 9.2.214, OriginLab Corp., Northampton, MA, USA) with repeated measures. Statistical significance was set at the level of 5% (*p* < 0.05).

The metabolic pathways of carotenoid and isoprenoid quinones biosynthesis have been generated using collection of tools for KEGG (Kyoto Encyclopedia of Genes and Genomes) mapping (http://www.genome.jp/kegg/mapper.html). KEGG is a database resource for understanding high-level functions and utilities of the biological system, such as the cell, from molecular-level information [47,48]. Compounds identified by chromatographic analysis were found in the database and received access numbers used to create maps and pathways. Access numbers: zeaxanthin (C06098), neoxanthin (C08606), phytofluene (C05414), neurosporene (C05431), β-carotene (C02094), α-carotene (C05433), canthaxanthin (C08583), myxoxanthophyll (C15941), astaxanthin (C08580), ubiquinone-10 (C11378), and menaquinone (C00828).

## 4. Conclusions

We have shown that beverage-spoiling bacteria *Asaia* spp. are able to synthesize a broad spectrum of carotenoids with relatively high concentration. In general, we have identified ten carotenoids and three isoprenoid quinones produced by *Asaia lannensis* and *A. bogorensis*. Our results show that *A. lannensis* strains are characterized by the biosynthesis of a wider range of carotenoids with statistically higher concentration. Similar conclusions were noted in the case of ubiquinone-10, menaquinone-7, and menaquinone-8 biosynthesis. It has also been found that one of the tested strains exhibits the ability to form extracellular carotenoids. This phenomenon is unusual, not only for *Asaia* spp., but also for other bacteria. Due to these bacteria being wide-spread, not only in functional beverages but also in natural environments, such as flowers, fruit, or gut and reproductive tract of mosquitos, it can be assumed that carotenoids can play an important role in adaptive properties to these environments. However, it is necessary to carry out further research on the properties of the compounds produced, as well as detailed research on metagenomics of these bacteria.

## Figures and Tables

**Table 1:** Identification of carotenoids and other secondary metabolites from *Asaia bogorensis* (ISD1, ISD2 and FFMW) and *Asaia lannensis* (IFCW, FMW1 and W4) based on ultrahigh-performance liquid chromatography (UHPLC), retention time (t_R_), UV–vis spectroscopic characteristics (λ_max_), and MS/MS spectroscopic pattern. Values are means of three determinations ± standard deviation. Concentration reported as µg per 100 mL of culture medium. nd—not detected.

Peak No.	t_R_ (min)	λ_max_ (nm)	[M − H]^+^ *m*/*z*	MS/MS Fragments	Compound	Formula	Classification	Compounds Concentration (µg/100 mL)					
								ISD1	ISD2	FFMW	IFCW	FMW1	W4
**1**	2.62	413, 438, 470	569.4788	551, 369	Zeaxanthin	C_40_H_56_O_2_	Carotenoids	nd	6.6 ± 0.09	8.5 ± 0.19	nd	nd	13.5 ± 0.61
**2**	3.58	286, 484, 520	601.5769	583	Neoxanthin	C_40_H_56_O_4_	Carotenoids	nd	nd	nd	nd	nd	2.7 ± 0.19
**3**	4.76	280	543.1193	381	Phytofluene	C_40_H_62_	Carotenoids	nd	nd	nd	nd	8.3 ± 0.22	21.3 ± 1.21
**4**	6.62	310, 486, 523	538.5083	95	Neurosporene	C_40_H_58_	Carotenoids	34.6 ± 2.08	nd	9.1 ± 0.44	19.1 ± 1.63	52.9 ± 2.59	50.9 ± 3.97
**5**	9.60	320, 486, 523	537.5193	519	α-Carotene	C_40_H_56_	Carotenoids	nd	nd	nd	nd	10.4 ± 0.83	14.9 ± 0.63
**6**	13.87	320, 486, 523	565.5489	532	Canthaxanthin	C_40_H_52_O_2_	Carotenoids	9.0 ± 0.36	nd	nd	2.4 ± 0.07	16.2 ± 0.77	8.7 ± 0.23
**7**	14.88	486	589.3875	439, 163	Synechoxanthin	C_40_H_42_O_4_	Carotenoids	nd	nd	nd	nd	3.0 ± 0.26	nd
**8**	15.72	300, 486, 509	758.4098	728, 705, 685, 633	Myxoxanthophyll	C_46_H_66_O_8_	Carotenoids	6.1 ± 0.37	1.2 ± 0.03	3.2 ± 0.18	2.8 ± 0.17	20.6 ± 1.56	5.0 ± 0.45
**9**	18.34	486	597.5089	579, 379, 285	Astaxanthin	C_40_H_52_O_4_	Carotenoids	3.0 ± 0.09	nd	nd	nd	3.9 ± 0.34	4.3 ± 0.27
**10**	20.07	286	651.5496	633, 397, 369, 351, 333	Menaquinone-7	C_46_H_64_O_2_	Isoprenoid quinones	3.3 ± 0.12	4.5 ± 0.21	3.2 ± 0.17	4.7 ± 0.22	9.8 ± 0.32	7.7 ± 0.23
**11**	21.14	450, 470, 486	537.3791	444, 177	β-Carotene	C_40_H_56_	Carotenoids	0.1 ± 0.01	0.7 ± 0.03	nd	nd	nd	nd
**12**	22.56	286	717.4471	575, 187	Menaquinone-8	C_51_H_72_O_2_	Isoprenoid quinones	nd	nd	0.5 ± 0.01	1.0 ± 0.02	4.5 ± 0.43	3.0 ± 0.12
**13**	24.92	280	863.6652	197	Ubiquinone-10	C_59_H_90_O_4_	Isoprenoid quinones	13.4 ± 0.60	22.5 ± 1.18	21.6 ± 0.95	38.1 ± 2.23	48.6 ± 4.43	6.3 ± 0.25
							**Total Content**	69.6 ± 3.61	35.5 ± 1.55	46.1 ± 1.94	68.1 ± 4.34	178.2 ± 11.74	138.3 ± 8.15
